# Skin bacteria of rainbow trout antagonistic to the fish pathogen *Flavobacterium psychrophilum*

**DOI:** 10.1038/s41598-021-87167-1

**Published:** 2021-04-06

**Authors:** Mio Takeuchi, Erina Fujiwara-Nagata, Taiki Katayama, Hiroaki Suetake

**Affiliations:** 1 Biomedical Research Institute, National Institute of Advanced Industrial Science and Technology (AIST), 1-8-31 Midorigaoka, Ikeda, Osaka 563-8577 Japan; 2 Faculty of Agriculture, Kindai University, 3327-204 Nakamachi, Nara, Nara 631-8505 Japan; 3 Institute for Geo-Resources and Environments, National Institute of Advanced Industrial Science and Technology (AIST), 1-1-1 Higashi, Tsukuba, Ibaraki 305-8567 Japan; 4 Faculty of Marine Science and Technology, Fukui Prefectural University, 1-1 Gakuen-cho, Obama, Fukui 917-0003 Japan

**Keywords:** Applied microbiology, Microbial communities

## Abstract

Rainbow trout fry syndrome (RTFS) and bacterial coldwater disease (BCWD) is a globally distributed freshwater fish disease caused by *Flavobacterium psychrophilum*. In spite of its importance, an effective vaccine is not still available. Manipulation of the microbiome of skin, which is a primary infection gate for pathogens, could be a novel countermeasure. For example, increasing the abundance of specific antagonistic bacteria against pathogens in fish skin might be effective to prevent fish disease. Here, we combined cultivation with 16S rRNA gene amplicon sequencing to obtain insight into the skin microbiome of the rainbow trout (*Oncorhynchus mykiss*) and searched for skin bacteria antagonistic to *F. psychrophilum*. By using multiple culture media, we obtained 174 isolates spanning 18 genera. Among them, *Bosea* sp. OX14 and *Flavobacterium* sp. GL7 respectively inhibited the growth of *F. psychrophilum* KU190628-78 and NCIMB 1947^T^, and produced antagonistic compounds of < 3 kDa in size. Sequences related to our isolates comprised 4.95% of skin microbial communities, and those related to strains OX14 and GL7 respectively comprised 1.60% and 0.17% of the skin microbiome. Comparisons with previously published microbiome data detected sequences related to strains OX14 and GL7 in skin of other rainbow trout and Atlantic salmon.

## Introduction

An increasing global population has caused a concomitant increased demand for food. Aquaculture is the world's fastest growing food production sector^1^, partly fulfilling the food demand. Infectious diseases are among the most pressing concerns in aquaculture development. For example, both rainbow trout fry syndrome (RTFS) and bacterial coldwater disease (BCWD) caused by *Flavobacterium psychrophilum*^2,3^ have become major causes of economic losses in salmonid fish such as rainbow trout (*Oncorhynchus mykiss*), coho salmon (*O. kisutsh*), and ayu (*Plecoglossus altivelis*) aquaculture worldwide. Fish diseases are usually controlled by antibiotics^4^, but antimicrobial resistance is a threat to the treatment worldwide. In fact, the World Health Organization (WHO) included antimicrobial resistance as one of the top 10 threats to global health^5^. Hence, a "One Health" approach, including aquaculture, is required to tackle antimicrobial resistance^6^. Vaccination can also prevent many bacterial diseases of fish. However, many problems need to be overcome before an effective vaccine for *F. psychrophilum* can become commercially available^7^.

Biological control is a possible alternative to antibiotics or vaccines^8^. *Carnobacterium* sp.^9^, *Pseudomonas* sp.^10–13^ and others^14–17^ have been identified as antagonistic against *F. psychrophilum*. Thus, these bacteria have been applied as functional feeds based on the notion that they would function in the fish gut^18^. The survival of rainbow trout fed with antagonistic strains and challenged with *F. psychrophilum* increased to some extent^10,11,17^ (relative percent survival values ranged from 28 to 58%). However, the practical application of this strategy has not yet proven successful. Recently, Nakashima et al.^19^ found that microbes in the digesta of ray-finned fish are separated from the epithelium by chitinous membranes that are not found in mammals. Thus, the effects of probiotics targeting the gut microbiome of fish may differ from that in mammals.

Skin damage is considered to create new portals for *F. psychrophilum* entry^20^. Therefore, manipulation of the skin microbiome, such as increasing the abundance of specific antagonistic bacteria against pathogens might be effective. Such bacteria should be residing on the skin mucus rather than the gut of fish. Boutin et al.^15^ and De la Fuente et al.^16^ isolated skin bacteria and searched for antagonists to *F. psychrophilum*. However, the isolated strains represented only 0.03%–1.8% of the total microbiota in the skin mucus^15^, or their population was not determined^16^. Recently, culturomics approach is used to understand gut microbiome of human^21^. Similarly, more effort should be made to isolate diverse bacteria from fish skin for further understanding of skin microbiome and the screening of antagonistic bacteria.

The present study aimed to analyze the skin microbiome of rainbow trout in order to culture a range of diverse bacteria for screening their antagonistic properties. Previous studies of isolating bacteria from fish skin have used only one or two complex media^15,22–30^. Here, we used 10 culture media containing different carbon sources or solidifying agents to isolate more diverse bacteria from the skin mucus of the rainbow trout. We then evaluated the antagonistic ability of the isolates against four strains of *F. psychrophilum*. The fraction of isolated strains in microbial communities of skin, gill, and the gut of rainbow trout, and surrounding water was estimated by high throughput sequencing analysis. We also compared our findings with published microbiome data of fish skin^29,31,32^ to see whether similar sequences to the cultured isolates were found previously.

## Results

### Strains isolated from skin mucus

**Figure 1:**
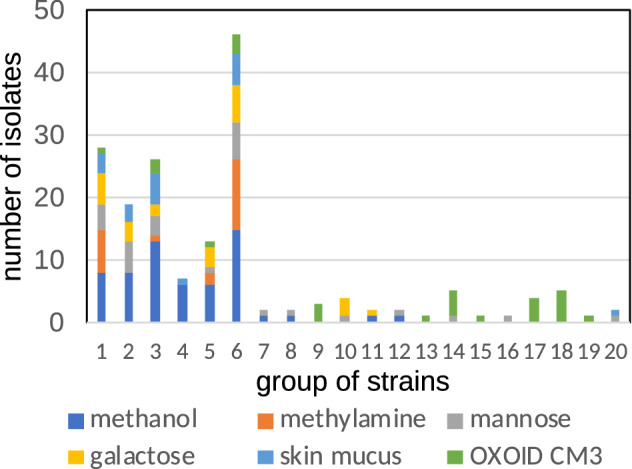
Numbers of isolates classified for strain group, composition of culture media.

We used culture media containing 6 different carbon sources. Sugars were included because lectin, a major component of fish mucus, binds various sugars^33^. Methanol and methylamine were included to isolate methylotrophs that was dominant in the skin of brook charr and is considered to compete with *F. psychrophilum*^34^. We obtained 174 isolates in total (Supplementary Table S1 online), and sequenced the 16S rRNA gene of the isolated strains. Based on the sequence identity of > 99% (considered to belong to the same species), the isolates were assigned to 20 groups spanning 18 genera and five phyla. Previous reports^35,36^ showed that closely related strains (including those shared > 99.2% of 16S rRNA gene similarity^36^) similarly exhibited antagonistic activities. In this study, a representative strain was selected from each group assuming that strains of each group exhibit similar antagonistic characteristics. Six of the 20 groups contained isolates that were retrieved only from OXOID CM3 medium and nine that were retrieved from media other than OXOID CM3 (Fig. 1). Group No. 6 represented by strain GL29 was the dominant and this group was obtained by all of the culture medium used (Fig. 1).

Representative strains in each group (Table 1) were further sequenced to obtain the almost complete 16S rRNA gene sequence (from 1392 to 1475 bp). These representative strains were used for further analyses. Strains OX11, OX32, and MN7 were closely related to possible pathogenic bacteria (*Pseudomonas fluorescens*, *Bacillus cereus*, and *Aeromonas caviae*, respectively) and were excluded from further analyses because it will not be practical to use these strains in future biological treatment (Table 1).

**Table 1:** Representative strains and closest species.

Group no	Representative strain	Closest species	Phylum	Sequence length	Identity (%)	Antagonistic
1	AG1	*Cellvibrio fibrivorans*	*Proteobacteria*	1440	99.0	
2	MN22	*Cellvibrio gandavensis*	*Proteobacteria*	1475	98.9	
3	TLA9	*Rheinheimera texasensis*	*Proteobacteria*	1424	99.7	+
4	TLA11	*Duganella ginsengisoli*	*Proteobacteria*	1454	98.4	+
5	AG5	*Pseudaeromonas pectinilytica*	*Proteobacteria*	1441	98.8	
6	GL29	*Acinetobacter tjernbergiae*	*Proteobacteria*	1439	99.9	+
7	TLA8	*Rhodococcus qingshengii*	*Actinobacteriota*	1420	99.9	+
8	MN10	*Deinococcus aquaticus*	*Deinococcota*	1402	99.4	+
9	OX16	*Deefgea chitinilytica*	*Proteobacteria*	1432	100.0	+
10	GL27	*Rhodoferax ferrireducens*	*Proteobacteria*	1441	98.1	
11	GL7	*Flavobacterium tructae*	*Bacteroidota*	1411	98.9	+
12	MN5	*Flavobacterium succinicans*	*Bacteroidota*	1429	98.4	
13	OX15	*Pelomonas saccharophila*	*Proteobacteria*	1432	98.6	+
14	OX14	*Bosea lupine*	*Proteobacteria*	1392	99.6	+
15	OX5	*Vogesella perlucida*	*Proteobacteria*	1443	99.9	
16	MN24	*Hydrogenophaga palleronii*	*Proteobacteria*	1439	98.7	
17	OX13	*Paenisporosarcina quisquiliarum*	*Firmicutes*	1460	100.0	
18	OX11	*Pseudomonas fluorecens*	*Proteobacteria*	1430	98.9	NE
19	OX32	*Bacillus cereus*	*Firmicutes*	1439	100.0	NE
20	MN7	Aeromonas rivipollensis*	*Proteobacteria*	1434	100.0	NE

Identity (%), 16S rRNA gene similarity with closest species; NE, not evaluated; Sequence length, length of 16S rRNA gene.

NE, not evaluated; Sequence length, length of 16S rRNA gene.

+ , postitive antagonistic action against at least one strain of *F. psychrophilum.*

* Strain MN7 was also close to *Aeromonas caviae* at sequence identity of 99.9%.

### Growth of the isolates on FLP, OXOID CM3, LB, and galactose containing medium

In order to assess whether our culture strategy was effective in culturing diverse skin bacteria, we examined the ability of the isolates to grow in four media. Strains TLA9, OX16, and OX5 could not grow in galactose containing medium, but thrived in OXOID CM3 and LB media (Supplementary Table S2 online). In contrast, strains AG1, MN22, and AG5 grew in galactose containing medium, but not in OXOID CM3 and LB media. All representative strains grew in FLP medium.

### Screening of antagonistic activity against *F. psychrophilum*

**Figure 2:**
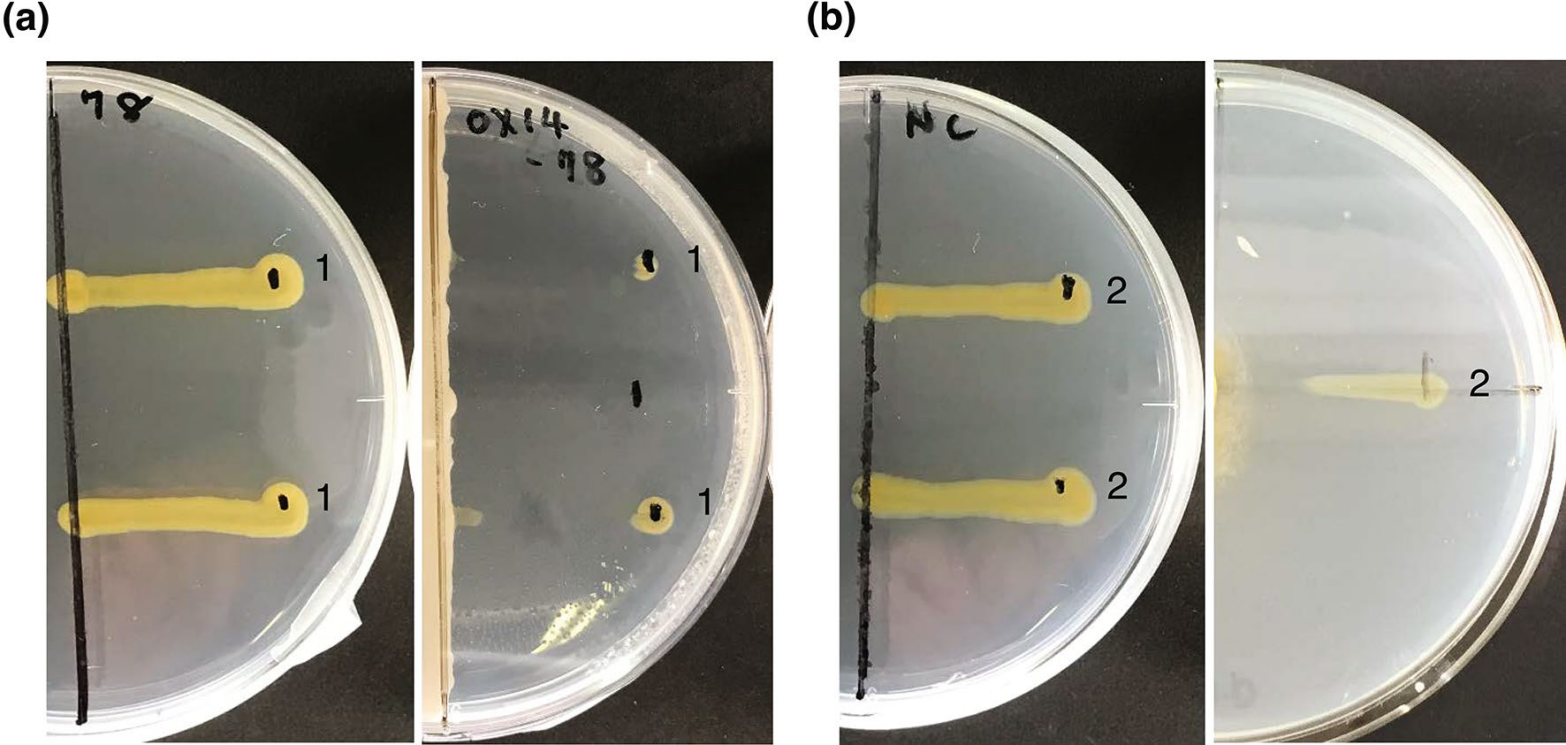
Cross-streaking method. The vertical line (**a**) and circle (**b**) in the right image is (**a**) strain OX14 and (**b**) GL7, and the lines marked with numerical digits are 1: *F. psychrophilum* KU190628-78 and 2: *F. psychrophilum* NCIMB 1947T. Left images are positive controls without antagonistic strains.

Culture supernatant of the 17 representative strains were added to the FLP medium inoculated with *F. psychrophilum* strains, and the growth of *F. psychrophilum* strains were monitored. Nine out of the 17 strains exerted antagonistic action against at least one *F. psychrophilum* strain (Supplementary Fig. S1 online, Table 1). Among the nine antagonistic strains, OX14 and GL7 inhibited the growth of *F. psychrophilum* KU190628-78 and NCIMB 1947^T^ to < 60%, respectively (Supplementary Fig. S1 online). The antagonistic activities of these two strains were also confirmed using the cross-streaking method (Fig. 2). Therefore, we further characterized antagonistic activity of strain OX14 against *F. psychrophilum* KU190628-78 and that of strain GL7 against *F. psychrophilum* NCIMB 1947^T^.

### Characteristics of antagonistic actions of strains OX14 and GL7 against *F. psychrophilum*

The pH of the culture supernatant of strain OX14 and GL7 were 7.6 and 7.2, respectively, and thus, the effect of pH change on the growth of *F. psychrophilum* strains was negligible. The CAS assay of siderophores was positive for strain GL7 as indicated by the color change of the medium, and weakly positive for strain OX14, suggesting the possibility that the antagonistic ability of these strains may be associated with siderophore production (Supplementary Fig. S2 online).

*Rheinheimera* sp. generates hydrogen peroxide which oxidizes L-lysine to produce an antimicrobial macromolecule, L-lysin oxidase^37^. Therefore, production of hydrogen peroxide was also assayed by a modified Prussian blue method. Both of strain OX14 and GL7 were negative for hydrogen peroxide production.

**Figure 3:**
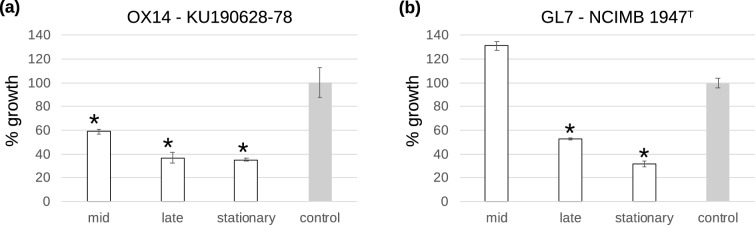
Antagonistic activity of bacterial culture supernatants against *F. psychrophilum* at various growth phases*.* Growth activities of (**a**) OX14 and (**b**) GL7 strains against *F. psychrophilum* KU190628-78 and NCIMB 1947^T^, respectively, at mid and late exponential growth, and stationary phases. Growth of positive control, in the absence of the supernatant, is taken as 100% (OD values of positive controls are 0.2 for KU190628-78 and NCIMB 1947^T^). Bars, standard deviation of triplicate samples. *Statistically significant decrease (*p* < 0.05).

The culture supernatant of strain OX14 inhibited the growth of *F. psychrophilum* KU190628-78 compared to the positive control experiment without culture supernatant. The growth inhibition was more effective during the late and stationary growth phases (35–37% of the control; Fig. 3a) compared to the mid growth phase (59% of the control; Fig. 3a). The culture supernatant of strain GL7 inhibited the growth of *F. psychrophilum* NCIMB 1947^T^, and was effective at the late exponential growth and stationary phases (32–53% of the positive control; Fig. 3b).

**Figure 4:**
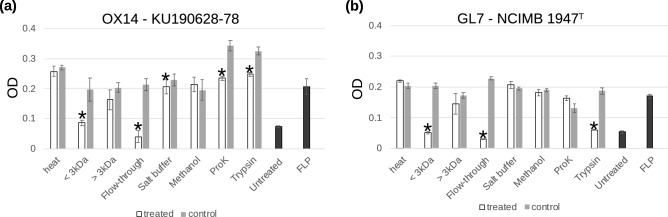
Antagonistic activity of heated, fractionated, and enzymatically digested bacterial culture supernatants against *F. psychrophilum*. Growth of (**a**) *F. psychrophilum* KU190628-78 and (**b**) *F. psychrophilum* NCIMB 1947^T^ incubated with culture supernatant of strain OX14 and GL7, respectively (white bars). Growth of *F. psychrophilum* strains with FLP medium similarly treated is used as a positive control (gray bars). Flow-through, flow-through from the anion exchange column. Salt buffer, eluate by high salt buffer. Methanol, eluate by methanol containing 1% formic acid. Growth of *F. psychrophilum* in untreated culture supernatant is presented as Untreated, and that in FLP medium is presented as FLP. Bars, standard deviation of the triplicates. *Statistically significant decrease.

Further analysis revealed that the antagonistic compounds produced by strains OX14 and GL7 were < 3 kDa in size and lost their activities after heating at 121 °C for 20 min (Fig. 4a,b). The antagonistic activity was lost in the supernatant of strain GL7 after proteinase K digestion (Fig. 4b). The culture supernatant of strain OX14 treated by proteinase K and trypsin inhibited the growth of *F. psychrophilum* compared to similarly treated control, but the activity was considered to be at least weakened by the treatment because OD values for these two were higher than those of *F. psychrophilum* in non-treated FLP medium (Fig. 4a). Fractions eluted with high salt buffer and methanol, which was considered to contain negatively charged proteins and small molecules, respectively, did not, or only slightly, inhibit the growth of *F. psychrophilum* (Fig. 4a,b). In contrast, the flow-through of the anion exchange column retained the antagonistic actions, suggesting that antagonistics were positively charged or neutral compounds at neutral pH. In order to further confirm antagonistic activity, culture supernatants were diluted 5- and tenfold with FLP medium, and growth inhibition against *F. psychrophilum* strains was examined. Five- and tenfold dilutions of the culture supernatant of strain OX14 did not inhibit the growth of *F. psychrophilum* KU190628-78. Five-fold dilution of the culture supernatant of strain GL7 inhibited the growth of *F. psychrophilum* NCIMB 1947^T^ slightly (to 97.4%), but no inhibition was observed when a tenfold dilution was used. In contrast, a tenfold concentrate of the culture supernatant of strain OX14 and GL7 obtained by freeze-drying inhibited growth of *F. psychrophilum* to 3% of the control.

### Microbial communities in rainbow trout and surrounding water

Supplementary Table S3 and Fig. S3 online show the general parameters of microbial communities characterized by 16S rRNA amplicon sequences. The number of reads that were finally used for analysis ranged between 30,203 and 44,849. The average numbers of OTUs (Sobs) per sample were 35, 69, and 380 for gill, the gut, and skin mucus, respectively, and 472 in the water sample. Skin and water shared 156 common OTU, whereas gill and the gut shared 1 and 4 OTU with water, respectively (Supplementary Fig. S4 online).

**Figure 5:**
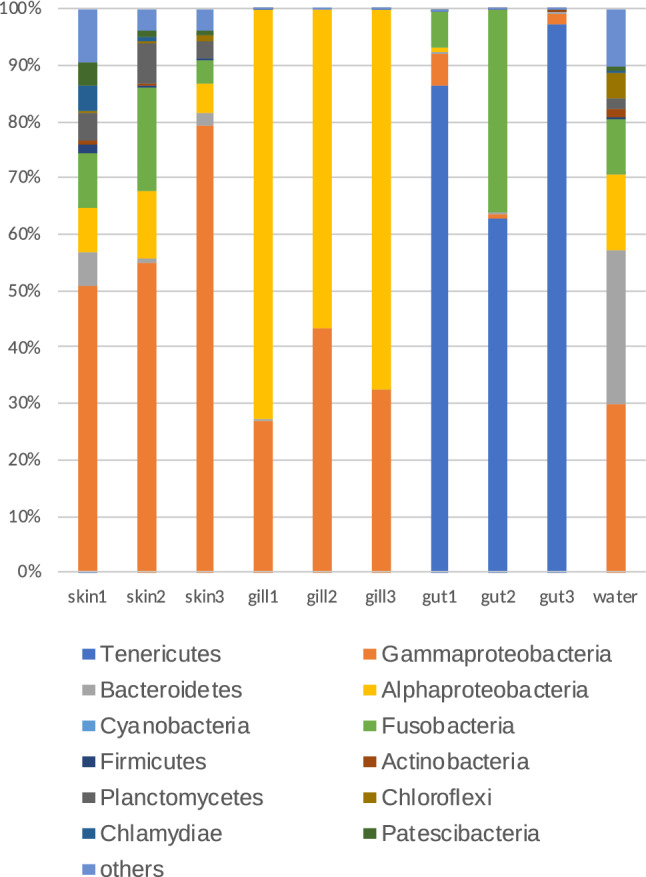
Relative abundance of phyla at sampling sites, and in individual fish, and rearing water.

Figure 5 shows that Proteobacteria dominated (43%–100%) of the microbial communities in skin, gill, and water. Among the Proteobacteria, the genus *Ralstonia* (gamma-Proteobacteria) predominated the microbial communities in skin (32–68%), whereas the genus *Bosea* (alpha-Proteobacteria) dominated those in the gill (57–74%). The family Comamonadaceae was a major component of the Proteobacteria in water. The phyla, Fusobacteria and Planctomycete were also found in skin mucus microbiomes (Fig. 5). The dominant phylum (63–97%) in the gut microbiome was Tenericutes (Fig. 5), which comprised one OTU belonging to the genus *Mycoplasma*. Others included Fusobacteria, i.e. *Cetobacterium somerae* at the species level, followed by Proteobacteria.

We compared our findings with two previous reports on the rainbow trout microbiome^29,31^. Proteobacteria was the dominant in skin microbiome of all of the studies. However, we found a difference in the major members next to Proteobacteria between the skin microbiomes in the present study and that described by Lowrey et al.^29^ and Terova et al.^32^. For example, next to Proteobacteria, Fusobacteria and Planctomycetes dominated in the present study (Fig. 5), whereas Bacteroidetes and Firmicutes were major phyla in Lowrey et al.^29^, and Tenericutes and Firmicutes in Terova et al.^32^. In contrast, the microbial communities in the gut were more similar, with the dominant family being Mycoplasmataceae (Tenericutes) in all of the studies.

### Fraction of isolated strains in skin microbial communities of fish

Based on OTU clustering of > 99% sequence similarity, sequences related to the cultured isolates represented 4.95%, 61.41%, 0.01%, and 10.03% of the microbial communities in skin, gill, the gut, and water, respectively (Table 2). On average, OTU similar to strain OX14 and GL7 accounted for 1.60% and 0.17% in skin microbial community, respectively. Sequences related to strain OX14 were the dominant in skin and gill microbial communities (Table 2).

**Table 2:** Abundance (percentage) of each isolate in microbial communities of rainbow trout (in this study).

Isolate	Skin-average (n = 3)	Gill-average (n = 3)	Gut-average (n = 3)	Water (n = 1)
AG1	*0.06*	*0.01*	0.00	*0.03*
MN22	*0.30*	0.00	0.00	*0.57*
TLA9	*0.16*	0.00	0.00	*0.18*
TLA11	*0.94*	0.00	0.00	*0.13*
AG5	*0.30*	0.00	0.00	0.00
GL29	*0.63*	0.00	0.00	*3.97*
TLA8	*0.02*	0.00	0.00	*0.10*
MN10	*0.06*	0.00	0.00	*0.08*
OX16	*0.04*	0.00	0.00	*0.42*
GL27	*0.01*	0.00	0.00	*0.70*
GL7	*0.17*	0.00	*0.01*	*0.11*
MN5	*0.00*	0.00	0.00	*0.14*
OX15	0.00	0.00	0.00	0.00
OX14	*1.60*	*61.40*	0.00	*0.04*
OX5	0.07	0.00	0.00	*0.02*
MN24	0.00	0.00	0.00	0.00
OX13	*0.12*	0.00	0.00	0.00
OX11	*0.43*	0.00	0.00	*3.35*
OX32	0.00	0.00	0.00	0.00
MN7	*0.02*	0.00	0.00	*0.04*
Total	4.95	61.41	0.01	10.03

Italics cells indicate sample with more than 0.01%.

We also investigated whether our isolates were among the skin microbial communities of fish in other studies. We detected OTUs similar to strains OX14 and GL7 at rates of 0.01% in Lowrey et al. (2015) (Supplementary Table S4), but abundance of OTU having > 99% similarity with all of our isolates were lower (0.02%) than that in our study (4.95%). While OTUs similar to 9 out of 20 isolates were detected at > 0.01% in skin microbial communities of rainbow trout in Terova et al.^32^ (0.54–0.88% in total), the proportions of sequences related to strain OX14 was low (0.004%), and those related to GL7 was not detected (Supplementary Table S5). We detected OTU having > 99% similarity with all of our isolates except MN5 and MN10 in the skin microbial community of Atlantic salmon^31^ (0.39–10.79% in total, Supplementary Table S6). The ratios of OTUs similar to strains GL7 and OX14 in the skin microbial communities were < 0.09% and < 0.04%, respectively.

## Discussion

We isolated 174 bacterial strains from the skin mucus of rainbow trout spanning 18 genera. These strains were estimated to account for 4.95% of the total bacterial community in skin, although it should be noted that this can be an overestimation considering the low taxonomic resolution of short-reads^38^. These values were higher than those of brook charr (*Salvelinus fontinalis*) using two culture media (0.03%–1.8%^15^). Spanggaard et al.^26^ isolated 1,018 bacteria belonging to nine genera from skin, gill, and the gut of rainbow trout using TSA medium. As shown in Supplementary Table S2 online, multiple culture media used for isolation in the present study would have facilitated the isolation of diverse bacteria from the skin mucus of rainbow trout. Among isolates, strain GL29, which was most close to *Acinetobacter tjernbergiae* was most frequently obtained (Fig. 1), and this was one of the most frequently detected groups in the skin microbial community (0.63%, Table 2). Although this group was not detected in rainbow trout in Lowrey et al.^29^, it was detected in skin of rainbow trout in Terova et al.^31^, and was the most frequently found group in Atlantic salmon skin (< 7.06%, Supplementary Table S6 online), suggesting that this group may be suitable to reside on skin of these fish. All of our strains grew on FLP medium, whereas several did not grow on OXOID CM3 and LB media (Supplementary Table S2 online). Hence, the FLP medium appears as a good alternative to the complex media commonly used for the isolation of relevant microorganisms in future studies. Our culture method expanded the range of culturable skin commensal bacteria that can be used to screen antagonistic bacteria against pathogens.

Strain OX14, which was closely related to *Bosea lupini*^39^, and strain GL7 that was most closely related to *Flavobacterium tructae*^40^ inhibited in vitro the growth of *F. psychrophilum*. They inhibited growth of *F. psychrophilum* to > 32% compared to the positive control, which was comparable to the growth inhibition by *Pseudomonas* sp.^12^*,* M174^10^, FF48^13^, and *Enterobacter* C6-6^17^ against *F. psychrophilum*. To date, reported antagonistic bacteria against *F. psychrophilum* originating from fish skin were restricted to the genus *Pseudomonas*^16^ retrieved from rainbow trout and to five genera (*Luteimonas*, *Microbacterium*, *Rhodococcus*, *Sphingopyxis*, and *Dietzia*) retrieved from brook charr^15^. The present study added potentially antagonistic bacteria belonging to the genera, *Flavobacterium* and *Bosea*. Sequences related to strains OX14 and GL7 were comprising of 1.60 and 0.17% of the skin microbial community. Furthermore, sequences related to strain OX14 was the dominant in the gill microbial community (61.4%) and also detected in rainbow trout used in previous studies^29,31^ although their population was lower compared to the present study. These strains may play a role in preventing *F. psychrophilum* at the surface of fish, the first line of defense, and could also be applicable in future biological treatment for fish diseases.

The production of siderophores^10^, bacteriocins^41^, lipoprotein^17^, hydrogen peroxide^35^, and blocking quorum-sensing^16^ are considered as the mechanisms underlying the antagonistic ability of bacteria. Among these, GL7 produced siderophores, whereas OX14 was also weakly positive. Pérez -Pascual et al*.*^42^ recently reported that *Flavobacterium* sp. strain 4466 inhibited the growth of *F. columnare* although the mechanism of the inhibition is not still clear. The mechanism of antagonistic activity against fish pathogens is considered to be different between strains 4466 and GL7 because culture supernatant of strain GL7 was effective but not of strain 4466. Zhang et al.^43^ reported that several strains of the genus *Bosea* produce the quorum quenching enzyme, AHL lactonase. The size of the lactonase identified in *Bosea* was 30 kDa^43^, which contradicts our finding that a < 3 kDa molecule of the culture supernatants suppressed the growth of *F. psychrophilum*. Flow-through of culture supernatants from anion exchange columns retained antagonistic activity, suggesting that antagonistic compounds are small and positively charged or neutral compounds. Future study will focus on identifying antagonistic compounds.

Four *F. psychrophilum* strains responded differently to the test strains. For example, OX14 and GL7 respectively inhibited only the growth of *F. psychrophilum* KU190628-78 and NCIMB 1947^T^. These variable responses might be due to strain differences, which is also associated with difficulties developing vaccines^7^. The population structure of *F. psychrophilum* has been classified by multilocus sequence typing (MLST)^44,45^, and sequence types are highly correlated to the host fish species. In this study, we used four strains having distinct sequence types; the sequence types of NCIMB 1947^T^ and SG950607 are CC-ST19^44^ and CC-ST 10^45^, respectively, and those of KU190628-78 and KU190628-79 are CC-ST48 and CC-ST52, respectively (unpublished data). This suggests that the different reactions might be related to their sequence types. Although some studies have examined the effects of antagonistic bacteria against multiple strains or serotypes of *F. psychrophilum*^10,12^, the effects of antagonistic bacteria on distinct *F. psychrophilum* sequence types remain to be thoroughly elucidated. It would be important to examine the relationship between *F. psychrophilum* sequence types and their reactions to antagonistic strains, and the mechanisms for variable responses among the strains before practical application. We believe that strains OX14 and GL7 could be applicable to ayu and coho salmon, respectively, in biological treatment for fish disease. The skin microbiomes of ayu and coho salmon have not been described, but strain OX14 and GL7 could be detected in the skin of Atlantic salmon (Supplementary Table S6 online), suggesting that these can thrive on the skin of other types of fish.

Because strains OX14 and GL7 are likely to reside on skin rather than the gut (Table 2), it would not be appropriate to applying them as feed additives. Instead, manipulating the skin microbiome to increase their abundance is promising. The skin microbiome of fish is known to be more influenced by surrounding water compared to the gut microbiome^32,46^, which was also suggested by our data (Supplementary Fig. S3 online). We also found that skin microbiomes of rainbow trout were not so similar between the present findings and those of others^29,31^ compared to the gut microbiomes, although it should be also noted that many of other factors can also leads to different results^47^. Therefore, the microbial community of fish skin might be controlled by changing the microbial community in surrounding water in a closed system. However, simply adding bacterial strains to rearing tanks did not alter the skin microbial community of brook charr^48^. In future, it is necessary to establish a technology to manipulate fish skin microbiome.

In conclusion, we isolated 20 skin bacteria estimated to comprise 4.95% of the skin microbial community of rainbow trout, and obtained two strains with antagonistic action against *F. psychrophilum*. These skin bacteria might serve as the basis for a novel technology with which to prevent fish diseases.

## Methods

### Sample collection

Rainbow trout were obtained from Fukui-Chuo-Uoichi co., ltd and maintained at 16 °C in 1,000-L tanks containing circulating groundwater at the Research Center for Marine Bioresources, Fukui Prefectural University, Japan for > 5 months. Three healthy fish weighing 356–420 g were randomly sampled using a sterilized net, anesthetized with 0.05% 2-phenoxyethanol (Wako, Osaka, Japan), then gently rinsed with sterile water. Skin mucus was sampled using a sterile glass slide from the whole left side of the fish. The first gill arch was cut using sterilized scissors. The contents of the aseptically obtained gut were washed out by gently rinsing with sterilized water, and the guts were stored on ice. Microorganisms in rearing water were also collected by filtering 600 mL of water through Millipore Express PLUS filters with 0.22-μm pores (Millipore Sigma Co., Ltd., Burlington, MA, USA). Samples were then brought to the laboratory on ice. Samples for DNA extraction were stored at − 80 °C. All experiments described above were conducted in compliance with the approved guidelines and regulations of Animal Care and Use Committee in Fukui Prefectural University. All procedures were approved by the same committee. Study design and reporting followed the ARRIVE guidelines.

### Isolation of bacteria from skin mucus

We used nitrate mineral medium^49^ with vitamin solution (biotin 20 μg L^−1^, folic acid 20 μg L^−1^, thiamine-HCl 20 μg L^−1^, B12 1 μg L^−1^, calcium pantothenate 50 μg L^−1^, riboflavin 50 μg L^−1^, nicotinamide 50 μg L^−1^) containing mannose (0.5%), galactose (0.5%), methanol (1% v/v), or methylamine (0.5%) as carbon sources, or containing autoclaved skin mucus (1% v/v). We also used OXOID CM3 medium as a representative complex medium. Either calcium (1.8 mM) or lanthanide (30 μM) was added as a co-factor for methanol dehydrogenase^50^ in methanol medium. Plates contained agar, or the agar substitute Gelrite (CP Kelco, Atlanta, GA, USA) with methanol and methylamine media. Half of the skin mucus was diluted 4 to sixfold with 0.2% saline, and spread on the surface of the medium, then the plates were incubated at 16 °C. The other half of the skin mucus was stored at − 80 °C for DNA extraction. After 5 days, colonies were subcultured on the same media. The original plates were further incubated and examined after 2 weeks of incubation. Newly emerged colonies were also picked and subcultured. One colony was transferred again onto the same medium to ensure a pure culture. At least 20 colonies were picked up from each plate (per carbon source) except for those of skin mucus that contained fewer colonies (Supplementary Table S1 online).

### 16S rRNA gene analysis of the isolates

Bacterial genomic DNA was extracted using phenol and chloroform as described by Cheng and Jiang^51^. The 16S rRNA gene was amplified by PCR using TaKaRa Taq Hot Star Version (Takara Bio Inc., Kusatsu, Japan) and the primer sets, Eub8F (5′-AGAGTTTGATCCTGGCTCAG-3′) and Univ1490R (5′-GGTTACCTTGTTACGACTT-3′) for bacteria^52^. The PCR reaction (10 μL) comprised 8 pmol of each primer, 1 μL of 10 × buffer, 0.8 μL of dNTP mixture, 0.05 μL of Takara Taq HS, and approximately 3 ng of genomic DNA and proceeded under the following conditions: 30 cycles at 98 °C for 10 s, 56 °C for 30 s, then 72 °C for 1.5 min. The PCR products were first sequenced with the universal primer 907R^53^. Sequence identities were calculated using ClustalX, and sequences with > 99% identity were considered as belonging to the same species and grouped accordingly. A representative strain was randomly selected from each group, and further analyzed. Almost the entire 16S rRNA gene in each representative strain was sequenced and compared with the GenBank database using BLAST (http://www.ncbi.nlm.nih.gov/BLAST/).

### Growth test with various media

The ability of isolates to grow on LB, OXOID CM3, FLP (complex media developed for *F. psychrophilum*^54^), and galactose containing media was assessed. Isolates were placed in 300 μL of each medium in 96 well plates and incubated at 16 °C for 2 weeks. Inoculated bacterial growth was periodically determined by measuring the OD at 630 nm using a Multiskan JX microplate reader (Thermo Labsystems Inc., Philadelphia PA, USA).

### Screening bacteria antagonistic to *Flavobacterium psychrophilum*

The *F. psychrophilum* strains, NCIMB 1947^T^ (isolated from *O. kisutch*), SG950607 (isolated from *O. mykiss*), KU190628-78 and KU190628-79 (isolated from *P. altivelis*) were the target pathogens. The growth of *F. psychrophilum* strains in FPL medium containing one-fifth volume of culture supernatants of the test strains was measured using microplates. All test strains except M22 were cultured in FLP medium until the early stationary phase. Strain M22 was cultured with galactose containing medium due to low growth yield in FLP medium. Culture supernatant obtained by centrifugation at 13,000 × g for 6 min, was passed through filters with 0.2-μm pores. *F. psychrophilum* strains were precultured in test tubes containing FLP medium at 16 °C for 2 days (OD reaches 0.3–0.4). Microplate wells containing 50 μL of supernatant and 10 μL of *F. psychrophilum* culture in FLP medium (final volume, 250 μL) were incubated in triplicate at 16 °C for 2 days. The negative and positive controls were FLP medium without and with *F. psychrophilum* strains, respectively. The turbidity in each well was measured at 660 nm using a Wallac1420 ARVO MX/Light microplate reader (PerkinElmer Life and Analytical Sciences Inc., Waltham, MA, USA).

### Cross-streaking method

Cross-streaking method was used to further confirm the antagonistic activity of strains OX14 and GL7 to *F. psychrophilum* KU190628-78 and NCIMB 1947^T^, respectively. For this, suspensions of the pathogens were streaked at right angles to the line of the cultures of strains OX14 and GL7 previously grown on FLP plates for 7 days, and incubated at 16 °C for 2 days. Antagonism by strains OX14 and GL7 was indicated by an interruption in the growth of the pathogen.

### Characterization of antagonistic compounds

Some microorganisms produce siderophores to capture iron from environments, and this is one mechanism through which antagonistic bacteria inhibit the growth of pathogens^10,16^. We assessed siderophore production using chrome azurol S (CAS) assays as described by Machuca and Milagres^55^ with modification. A hole in the FLP medium with a diameter of 3 cm was aseptically punched, and CAS-blue agar was poured into it. Test strains were inoculated across the border of the two media and the plates were incubated at 16 °C for one week. The color change of the CAS-blue agar was considered as an indication of positive reaction.

Production of hydrogen peroxide was assayed by a modified Prussian blue method^56^ due the reactivity of the FLP medium with hexacyanoferrate(III). 1 g each of FeCl_3_·6H_2_O and potassium hexacyanoferrate(III) was dissolved in separate 50 mL of water. These solutions (50 μL) were added to 900 μL of water. Then, test solution of 50 μL was added to monitor the color change of the mixture after 5 min. This modified method was as sensitive as the original Prussian blue agar method (> 1 nmol of hydrogen peroxide). Strains OX14 and GL7 were grown in galactose containing medium, and the culture supernatants were used as test solutions.

To further characterize antagonistic compounds produced by strains OX14 and GL7, we assayed the antagonistic action of culture supernatants sampled at mid and late exponential, and stationary phases as described above. Culture supernatants were also assessed after heating at 121 °C for 20 min, fractionation with Centriprep YM-3 (Merck KGaA, Darmstadt, Germany) to collect compounds of < and > 3 kDa and following proteinase K and trypsin digestion. These enzymes in 20 mM Tris–HCl (pH 8.0) were added to the culture supernatant at a final concentration of 0.1 mg mL^−1^ and incubated at 37 °C for 3 h. Culture supernatants (6 mL) were loaded onto an Oasis MAX cartridge 3 cc (Waters Corp., Milford, MA, USA), and negatively charged proteins were eluted with 1 mL of high salt buffer (HEPES 50 mM, 0.5 M NaCl, pH 7.2). Other acidic small compounds were further eluted with 1 mL of methanol containing 1% formic acid^57^. For each treatment, FLP medium similarly treated was used as a positive control. Five- and tenfold-diluted supernatants as well as tenfold concentrate by freeze-drying were also assayed.

### Analysis of 16S rRNA amplicon sequences

We extracted DNA from skin mucus, gill, the gut (posterior gut) of rainbow trout, and the rearing water using the FastDNA SPIN Kit for Soil (MP Biomedicals Inc., Santa Ana, CA, USA) as described by the manufacturer. Gill samples were suspended in 0.2% saline, and homogenized using a sterile spatula. Large pieces of debris were removed by centrifugation at 300×g for 1 min, then microbial cells were collected by further centrifugation of the supernatant at 5000×g for 5 min. Microorganisms in the rearing water were resuspended in 5 mL of sterilized water, and collected by centrifugation at 5000×g for 5 min, then DNA was extracted. Archaea are minor components of the microbial community in fish skin^58^; thus, the V3–V4 region (approximately 450 bp) of the bacterial 16 S rRNA gene was amplified using PCR with primers 5′ ACACTCTTTCCCTACACGACGCTCTTCCGATCT- NNNNN- fwd_primer-3′ and 5′-GTGACTGGAGTTCAGACGTGTGCTCTTCCGATCT- NNNNN-rev_primer’, where fwd_primer is 341F^59^ and rev_primer is 805R^59^. PCR was performed with Good Taq (Intégrale Co., Ltd. Tokyo, Japan) under the following conditions: 95 °C for 10 min; 30 cycles at 95 °C for 30 s, 55 °C for 30 s, then 72 °C for 40 s. The PCR products were cleaned with AMPure XP (Beckman Coulter, Brea, CA) and used for indexing PCR. The PCR reaction (10 μL) comprised 5 pmol of each primer, 1 μL of 10 × Ex buffer, 0.8 μL of dNTP mixture, 0.1 μL of TaKaRa Ex Taq HS (Takara Bio Inc., Kusatsu, Japan), and 10 ng of the purified PCR product and proceeded under the following conditions: 94 °C for 2 min, 10 cycles at 94 °C for 30 s, 60 °C for 30 s, and 72 °C for 30 s, then 72 °C for 5 min. After another round of AMPure XP (Beckman Coulter, Brea, CA) clean-up, multiplexed amplicons were sequenced with Illumina MiSeq (Illumina, CA, 300 bp paired-end reads) at Seibutsu Giken Co., Ltd (Kanagawa, Japan).

The sequences were analyzed using mothur ver. 43^60^ as previously described^61^ with the following modifications. Chimeric sequences were removed using VSEARCH^62^. Singleton was removed to reduce error^63^. After clustering sequences into operational taxonomic units (OTUs) using the OptiClust algorithm^64^, each unique OTU was categorized by a Bayesian classifier and on the Silva taxonomy SSU Ref NR99 release 132 datasets^65^ with a confidence threshold of 80%. Taxonomic information of the OTUs (> 97% sequence similarity) was obtained from the majority consensus taxonomies of the sequences within the OTU with a consensus confidence threshold of 80%. We also conducted OTU clustering (> 99% sequence similarity) using Illumina sequencing reads and Sanger-based sequences of isolates to determine the abundance of isolates in the microbial communities. Data in Lowrey et al.^29^, Terova et al.^31^ and Webster et al.^32^ were also analyzed in this manner.

### Statistical analysis

Differences between positive controls and test samples were calculated using Student’s t tests^66^. Values with p < 0.05 were deemed statistically significant.

## Supplementary Information

Supplementary Information

## Data Availability

The 16S rRNA gene sequences of the isolates determined herein were deposited in the DDBJ/GenBank/EMBL database under accession numbers LC566124 –LC566143. Other sequence data generated in this study were deposited in the NCBI Sequence Read Archive under BioProject ID PRJDB10074.
