# Supplementary material for: Skin bacteria of rainbow trout antagonistic to the fish pathogen Flavobacterium psychrophilum
Source: Sci Rep. 2021 Apr 6;11:7518. doi: 10.1038/s41598-021-87167-1 (PMC8024317; doi:10.1038/s41598-021-87167-1)
Supplement: Supplementary file 1 — Supplementary Information [file 41598_2021_87167_MOESM1_ESM.pdf]

Supplementary Information

Skin bacteria of rainbow trout antagonistic to the fish pathogen *Flavobacterium psychrophilum*

Mio Takeuchi[1 *], Erina Fujiwara-Nagata[2], Taiki Katayama[3], Hiroaki Suetake[4]

1: Biomedical Research Institute, National Institute of Advanced Industrial Science and Technology (AIST), 1-8-31 Midorigaoka, Ikeda, Osaka 563-8577, Japan

2: Faculty of Agriculture, Kindai University, 3327-204 Nakamachi, Nara, Nara 631-8505, Japan

3: Institute for Geo-resources and Environments, National Institute of Advanced Industrial Science and Technology (AIST), 1-1-1 Higashi, Tsukuba, Ibaraki 305-8567, Japan

4: Faculty of Marine Science and Technology, Fukui Prefectural University, 1-1 Gakuen-cho, Obama, Fukui 917-0003, Japan

*: corresponding author: Mio Takeuchi

Biomedical Research Institute, National Institute of Advanced Industrial Science and Technology (AIST), 1-8-31 Midorigaoka, Ikeda, Osaka 563-8577, Japan

Tel: +81-29-861-2478, Fax: +81-72-751-9628, E-mail: takeuchi-mio@aist.go.jp

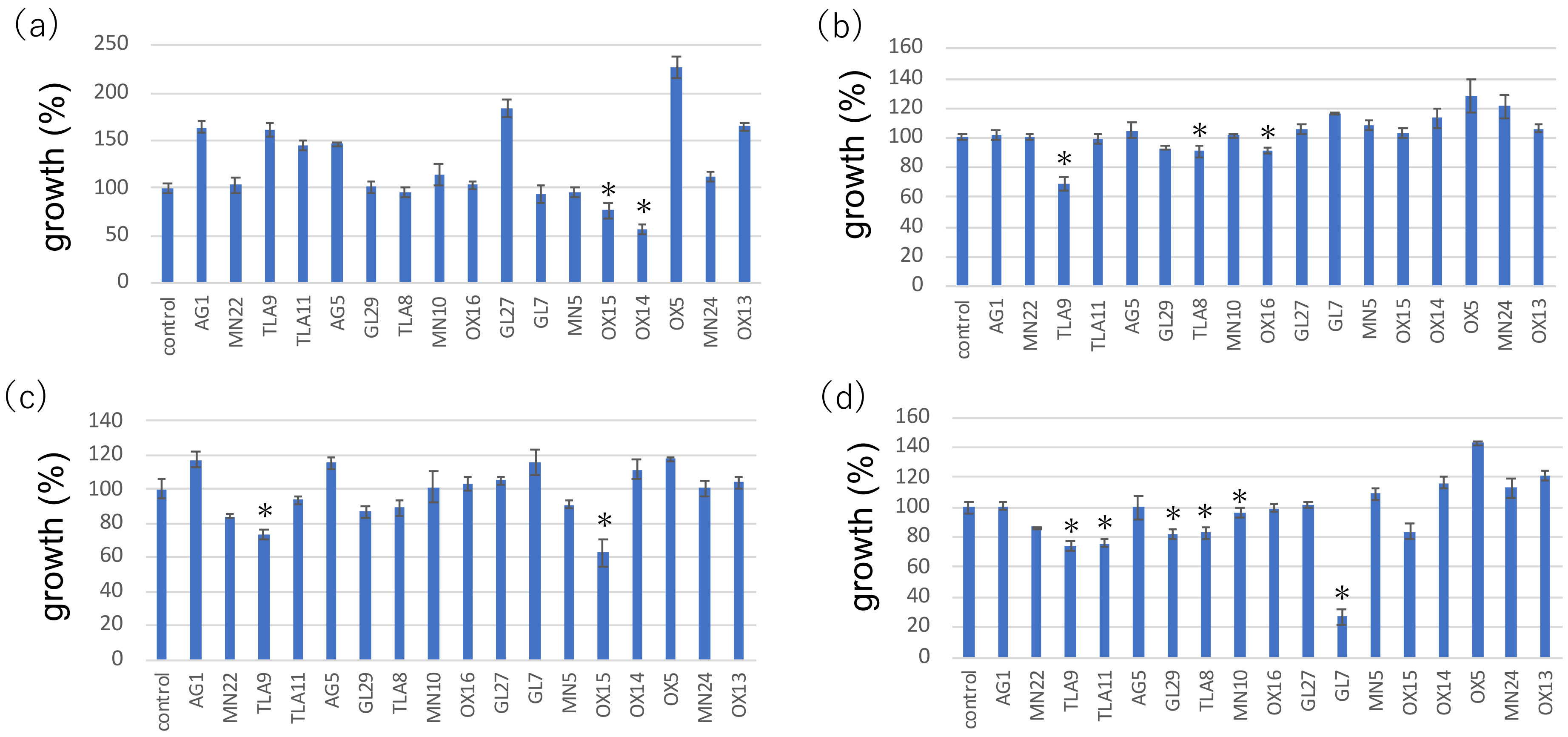

Figure S1. Growth inhibition of culture supernatant of 17 test strains against *F. psychrophilum* (a) KU190628-78, (b) KU190628-79, (c) SG950607, and (d) NCIMB 1947^T. Growth of *F. psychrophilum* without the culture supernatant of test strains is taken as 100%. Bars, standard deviation of the triplicates. *Statistically significant decrease.

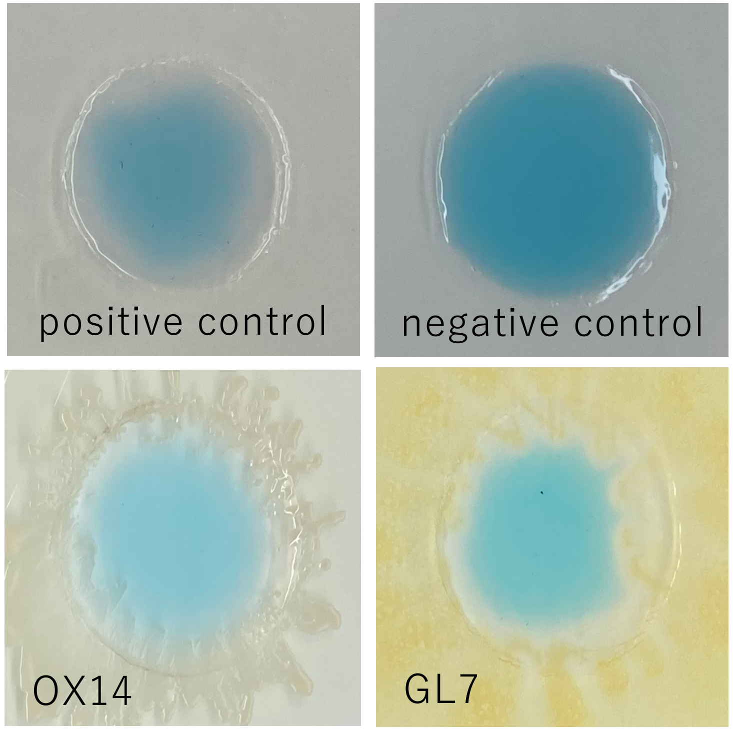

Figure S2. CAS assay with strain OX14 and strain GL7. 50mM EDTA spotted at the periphery of the CAS blue agar was used as a positive control. 50mM EDTA was used as a positive control.

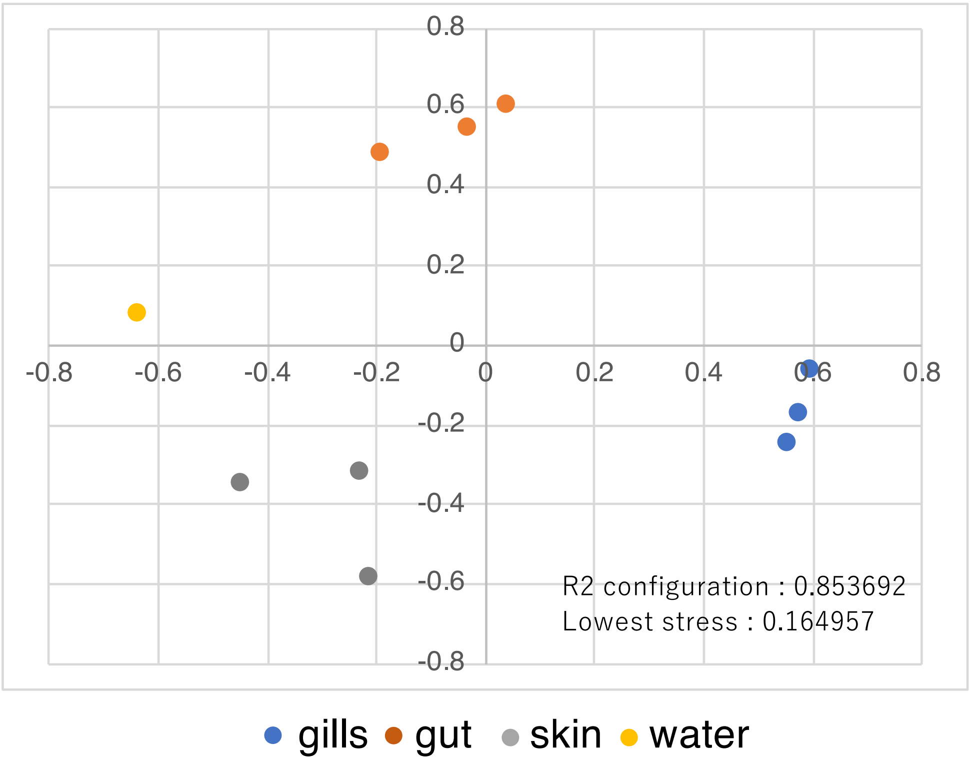

Figure S3. NMDS ordination analysis based on the Bray-Curtis index of OTUs in skin, gill, the gut and water microbiomes. NMDS, nonmetric multidimensional scaling.

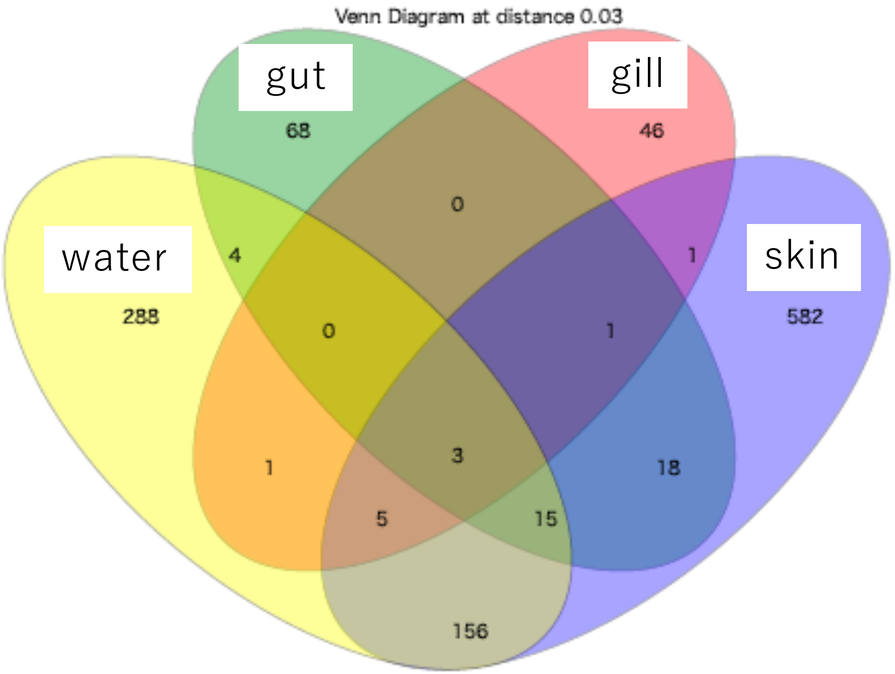

Figure S4. Venn diagram showing shared OTUs among skin, gill, and the gut of rainbow trout, and the rearing water.

Table S1. Number of isolates obtained from each growth media.

media	No. of isolates
OXOID CM3 (OX)	26
mannose (MN)	26
galactose (GL)	23
skin mucus (SM)	18
methylamine (gellrite) (AG)	11
(agar) (AA)	10
methanol Ca (gellrite) (TCG)	19
(agar) (TCA)	10
methanol La (gellrite) (TLG)	20
(agar) (TLA)	11
total	174

Table S2. Growth of representative strains on various media.

Isolates were placed in 300 µL of each medium in 96 well plates and incubated at 16°C for 2 weeks.

Group	representative strain	galactose containng medium	OXOID CM3	LB	FLP
1	AG1	+	-	-	+
2	MN22	+	-	-	±
3	TLA9	-	+	+	+
4	TLA11	+	-	+	+
5	AG5	+	-	-	+
6	GL29	±	+	+	+
7	TLA8	+	+	+	+
8	MN10	±	+	-	+
9	OX16	-	+	+	+
10	GL27	+	-	+	+
11	GL7	+	+	+	+
12	MN5	±	+	-	+
13	OX15	+	+	+	+
14	OX14	±	+	+	+
15	OX5	-	+	+	+
16	MN24	+	+	+	+
17	OX13	±	+	+	+

+, growth, -, no growth, ±, weak growth.

Table S3. General characteristics of amplicon sequence analysis.

	Sequences (n)	Good's coverage	Sobs (OTU)	Chao1	ACE	Shannon index	*Invsimpson	*Shannon evenness	*Simpson evenness
skin-1	35,013	0.998	584	673	612	3.87	8.20	0.610	0.014
skin-2	30,285	0.998	234	379	295	2.65	4.96	0.486	0.021
skin-3	30,203	0.998	323	408	371	2.16	2.27	0.374	0.007
gill-1	37,677	1.000	30	56	173	0.77	1.71	0.235	0.065
gill-2	38,086	1.000	39	44	45	0.79	2.02	0.220	0.055
gill-3	35,838	1.000	37	118	67	0.70	1.79	0.197	0.052
gut-1	41,536	1.000	73	98	88	0.70	1.34	0.167	0.020
gut-2	43,169	0.999	65	93	82	0.90	2.04	0.223	0.036
gut-3	44,849	1.000	69	75	73	0.25	1.07	0.061	0.017
water	41,538	0.999	472	480	474	4.65	41.00	0.757	0.088

*Invsimpson: Inverse of Simpson's diversity index.

*Shannon evenness: a Shannon index-based measure of evenness

*Simpson evenness: a Simpson index-based measure of evenness

Table S4. Abundance (%) of isolates found in microbial communities in rainbow trout (Lowrey et al. 2015).

Average values for skin (n=5), gill (n=6), anterior gut (n=5), and posterior gut (n=6) are presented.

Gray cells indicate sample with more than 0.01%.

Isolates	skin	gill	anterior gut	posterior gut
AG1	0.00	0.00	0.00	0.00
MN22	0.00	0.00	0.00	0.00
TLA9	0.00	0.01	0.00	0.00
TLA11	0.00	0.00	0.00	0.00
AG5	0.00	0.00	0.00	0.00
GL29	0.00	0.00	0.00	0.00
TLA8	0.00	0.00	0.00	0.03
MN10	0.00	0.00	0.00	0.00
OX16	0.00	0.00	0.00	0.00
GL27	0.00	0.00	0.00	0.00
GL7	0.01	0.00	0.00	0.00
MN5	0.00	0.00	0.00	0.02
OX15	0.00	0.00	0.00	0.00
OX14	0.01	0.00	0.00	0.00
OX5	0.00	0.00	0.00	0.00
MN24	0.00	0.00	0.00	0.00
OX13	0.00	0.00	0.00	0.00
OX11	0.00	0.00	0.00	0.00
OX32	0.00	0.01	0.00	0.00
MN7	0.00	0.00	0.00	0.00
**total**	**0.02**	**0.03**	**0.00**	**0.05**

Table S5. Abundance (%) of isolates found in microbial communities of rainbow trout (Terova et al. 2021).
Average values (n=6) are presented for each sample group.
Gray cells indicate sample with more than 0.01%.

Isolates	skin (TM0)	skin (TM100)	gut (TM0)	gut (TM100)
AG1	0.00	0.02	0.00	0.00
MN22	0.01	0.07	0.00	0.00
TLA9	0.00	0.00	0.00	0.00
TLA11	0.00	0.00	0.00	0.00
AG5	0.01	0.01	0.00	0.00
GL29	0.05	0.12	0.00	0.00
TLA8	0.01	0.00	0.00	0.00
MN10	0.00	0.00	0.00	0.00
OX16	0.03	0.09	0.00	0.00
GL27	0.00	0.00	0.00	0.00
GL7	0.00	0.00	0.00	0.00
MN5	0.13	0.10	0.00	0.00
OX15	0.00	0.00	0.00	0.00
OX14	0.00	0.00	0.00	0.00
OX5	0.00	0.00	0.00	0.00
MN24	0.00	0.00	0.00	0.00
OX13	0.00	0.00	0.00	0.00
OX11	0.24	0.26	0.00	0.00
OX32	0.00	0.00	0.00	0.00
MN7	0.05	0.20	0.00	0.07
**total**	**0.54**	**0.88**	**0.00**	**0.07**

Table S6. Abundance (%) of isolates in microbial communities of Atlantic salmon (Webster et al. 2018).
For skin samples, average values for CON (n=11), SAW (n=12),
SPY (n=12), TWD (n=12), FRM (n=12), MHV (n= 12), and CYN (n=11) are presented.
For gut samples, average values for CON (n=10), SAW (n=11), SPY (n=11),
SPY (n=11), TWD (n=12), FRM (n=12), MHV (n= 12), and CYN (n=13) are presented.
Gray cells indicate sample with more than 0.01%.

Skin

Isolates	CON	SAW	SPY	TWD	FRM	MHV	CYN
AG1	0.00	0.05	0.00	0.01	0.00	0.00	0.00
MN22	0.09	0.07	0.06	0.00	0.00	0.05	0.03
TLA9	0.00	0.03	0.08	0.03	0.02	0.14	0.00
TLA11	0.02	0.00	0.00	0.00	0.00	0.00	0.04
AG5	0.00	0.00	0.12	0.00	0.00	0.00	0.01
GL29	0.15	0.16	0.17	0.05	0.05	0.00	7.06
TLA8	0.00	0.05	0.21	0.07	0.03	0.13	0.03
MN10	0.00	0.00	0.00	0.00	0.00	0.00	0.00
OX16	0.00	0.00	0.00	0.01	0.00	0.00	0.00
GL27	0.07	0.01	0.22	0.21	0.14	0.02	0.09
GL7	0.00	0.00	0.09	0.00	0.00	0.06	0.00
MN5	0.00	0.00	0.00	0.00	0.00	0.00	0.00
OX15	0.02	0.23	0.08	0.06	0.23	0.03	0.05
OX14	0.00	0.04	0.00	0.00	0.00	0.00	0.00
OX5	0.00	0.00	0.22	0.07	0.00	0.00	0.00
MN24	0.00	0.03	0.02	0.02	0.00	0.06	0.00
OX13	0.00	0.00	0.09	0.04	0.02	0.00	0.00
OX11	0.00	0.00	0.01	0.00	0.00	0.21	0.11
OX32	0.02	0.06	0.00	0.00	0.00	0.00	0.02
MN7	0.01	0.01	0.13	0.08	0.09	0.26	3.35
**total**	**0.39**	**0.76**	**1.50**	**0.65**	**0.59**	**0.96**	**10.79**

Gut

Isolates	CON	SAW	SPY	TWD	FRM	MHV	CYN
AG1	0.00	0.00	0.00	0.00	0.00	0.00	0.00
MN22	0.00	0.00	0.00	0.00	0.00	0.00	0.02
TLA9	0.00	0.00	0.00	0.00	0.00	0.00	0.00
TLA11	0.00	0.00	0.00	0.00	0.00	0.00	0.00
AG5	0.00	0.00	0.00	0.00	0.00	0.00	0.24
GL29	0.00	0.00	0.00	0.00	0.00	0.00	0.59
TLA8	0.00	0.00	0.00	0.00	0.00	0.00	0.08
MN10	0.00	0.00	0.00	0.00	0.00	0.00	0.00
OX16	0.00	0.00	0.00	0.40	0.00	0.00	0.00
GL27	0.00	0.00	0.00	0.10	0.00	0.00	0.00
GL7	0.01	0.00	0.03	0.00	0.00	0.00	0.00
MN5	0.00	0.00	0.00	0.00	0.00	0.00	0.00
OX15	0.01	0.02	0.06	0.05	0.00	0.00	0.03
OX14	0.00	0.00	0.00	0.00	0.00	0.00	0.00
OX5	0.00	0.00	0.00	0.00	0.00	0.00	0.00
MN24	0.00	0.01	0.02	0.01	0.00	0.00	0.00
OX13	0.00	0.00	0.00	0.00	0.00	0.00	0.00
OX11	0.00	0.00	0.00	0.01	0.00	0.14	0.03
OX32	0.00	0.00	0.00	0.00	0.00	0.00	0.00
MN7	0.15	0.04	0.35	0.11	1.70	0.04	4.16
**total**	**0.17**	**0.07**	**0.46**	**0.68**	**1.72**	**0.19**	**5.15**

Water

Isolates	CON	MHV	CYN	SAW	SPY	TWD	FRM
AG1	0.00	0.00	0.00	0.00	0.00	0.01	0.02
MN22	0.13	0.19	0.04	0.10	0.10	0.01	0.00
TLA9	0.00	0.00	0.00	0.00	0.14	0.01	0.05
TLA11	0.00	0.00	0.00	0.00	0.00	0.00	0.00
AG5	0.01	0.05	0.00	0.00	0.00	0.00	0.00
GL29	0.58	0.01	0.01	0.20	0.09	0.02	0.00
TLA8	0.04	0.07	0.02	0.00	0.04	0.00	0.00
MN10	0.00	0.00	0.00	0.00	0.00	0.00	0.00
OX16	0.00	0.00	0.00	0.00	0.05	0.02	0.00
GL27	0.03	0.05	0.04	0.03	0.19	0.29	0.12
GL7	0.01	0.00	0.01	0.00	0.44	0.01	0.00
MN5	0.00	0.00	0.00	0.00	0.00	0.00	0.00
OX15	0.02	0.12	0.02	0.07	0.04	0.02	0.12
OX14	0.00	0.00	0.00	0.00	0.02	0.00	0.00
OX5	0.00	0.00	0.00	0.00	0.16	0.00	0.00
MN24	0.00	0.00	0.00	0.01	0.03	0.03	0.00
OX13	0.00	0.00	0.00	0.00	0.00	0.00	0.00
OX11	0.01	0.13	0.12	0.00	0.00	0.03	0.00
OX32	0.03	0.09	0.00	0.00	0.00	0.00	0.00
MN7	0.76	0.30	0.02	0.02	0.10	0.02	0.03
**total**	**1.63**	**1.01**	**0.27**	**0.42**	**1.41**	**0.48**	**0.37**

CON, Conservatoire National du Saumon Sauvage; CYN , NRW Cynrig Hatchery;
MHV, Marine Harvest Scotland; SAW, Towy; SPY, Spey; TWD, Tweed
